# Using NaOH@Graphene oxide-Fe_3_O_4_ as a magnetic heterogeneous catalyst for ultrasonic transesterification; experimental and modelling

**DOI:** 10.1038/s41598-024-64865-0

**Published:** 2024-06-22

**Authors:** Sepideh Moradi Haghighi, Alireza Hemmati, Hamidreza Moghadamzadeh, Ahad Ghaemi, Nahid Raoofi

**Affiliations:** 1 Department of Chemical Engineering, Faculty of Engineering, Islamic Azad University, South Tehran Branch, Tehran, Iran; 2 School of Chemical, Petroleum and Gas Engineering, Iran University of Science and Technology, (IUST), Narmak, Tehran, 16846 Iran

**Keywords:** Ultrasonic, Heterogeneous catalyst, Biodiesel, Kinetic study, Response surface methodology, Environmental sciences, Materials science, Mathematics and computing, Nanoscience and technology

## Abstract

Burning fossil fuels causes toxic gas emissions to increase, therefore, scientists are trying to find alternative green fuels. One of the important alternative fuels is biodiesel. However, using eco-friendly primary materials is a main factor. Sustainable catalysts should have high performance, good activity, easy separation from reaction cells, and regenerability. In this study, to solve the mentioned problem NaOH@Graphene oxide-Fe_3_O_4_ as a magnetic catalyst was used for the first time to generate biodiesel from waste cooking oil. The crystal structure, functional groups, surface area and morphology of catalyst were studied by XRD, FTIR, BET, and FESEM techniques. The response surface methodology based central composite design (RSM-CCD) was used for biodiesel production via ultrasonic technique. The maximum biodiesel yield was 95.88% in the following operation: 10.52:1 molar ratio of methanol to oil, a catalyst weight of 3.76 wt%, a voltage of 49.58 kHz, and a time of 33.29 min. The physiochemical characterization of biodiesel was based to ASTM standard. The magnetic catalyst was high standstill to free fatty acid due to the five cycle’s regeneration. The kinetic study results possess good agreement with first-order kinetics as well as the activation energy and Arrhenius constant are 49.2 kJ/min and 16.47 * 10^10^ min^−1^, respectively.

## Introduction

Rising crude oil prices, environmental pollutants from fossil fuels combustion, and their limited resources have led scientists to find clean renewable fuels like biodiesel^1^. Biodiesel is renewable fuel composed of a mixture of mono alkyl esters^2^. Biodiesel is usually produced from the transesterification of triglyceride oil with methanol. Biodiesel contains about 11% by weight of oxygen more than conventional diesel, so it has high energy production efficiency, it has no sulfur or its sulfur is much lower than commercial diesel, so there is no concern about corrosion of the internal surfaces of the engine. As well as, it has a high flash point (fire temperature), non-toxic, biodegradable and emits a small amount of CO_2_, CO… during combustion^3^.

The transesterification reaction (ester exchange) is a reversible reaction that involves the conversion of one ester to different esters^4^. In the biodiesel production process, ester exchange causes the conversion of triglycerides to methyl esters and glycerin. 80% of the price of biodiesel is related to raw materials especially primary feedstock oil. In the production of the biodiesel process, feedstock oils should be available and non-edible. Waste cooking oil was a kind of waste materials which it generates from restaurants, hospital, and café trial. Waste cooking oil is a good choice for biodiesel production because it has high amount of fatty acid esters and a relatively low cost for collection^5^.

The use of catalysts accelerates the rate of transesterification reaction^6^. Generally, the catalysts used for biodiesel production on an industrial scale are acidic and alkaline homogeneous and heterogeneous catalysts. The use of acidic homogeneous catalysts prevents the formation of soap and the homogeneous alkaline catalyst also has a high catalytic activity, but the biggest problem of using these acidic and alkaline homogeneous catalysts is the separation and purification of the catalyst from biodiesel as the final product. Heterogeneous catalysts are generally less sensitive to free fatty acids than homogeneous catalysts^7^, the use of heterogeneous acid catalysts that have the ability to recycle, their production is not economically viable and in some cases, washing of active sites leads to product contamination and reduced efficiency^8^. Alkaline heterogeneous catalyst, which is environmentally friendly and requires less time to produce than a similar acid catalyst, has been used. However, if some of this catalysts are exposed to the air, it is more likely to be toxic and due to the production of soap, therefore the biodiesel yield is significantly reduced^9^. Nano-catalysts have special properties such as their selective reactivity, high activation energy and controlled rate of reaction, easy recovery and recyclability, and easier separation from reaction mixture^10^. Rahman et al.^11^ used calcareous eggshell as a solid catalyst for biodiesel production. They impregnated different metal oxides on eggshell and produced biodiesel (~ 94.5%) at optimum conditions: 7 wt% catalyst weight, 12:1 methanol to molar ratio, and 180 min. Foroutan et al.^12^ used rice husk ash (RHA)/ CuO/K_2_CO_3_ as a catalyst for biodiesel production. They achieved maximum biodiesel yield 98.1% under optimum condition 3 wt% catalyst, reaction temperature of 65 °C, and 120 min. Ahranjani et al.^13^ synthesized magnetic carbon nanotubes doped cadmium oxide as a magnetic heterogeneous catalyst for biodiesel production.

Graphene oxide (GO) is widely used as catalyst support for heterogeneous catalyst due to its high thermal and mechanical stability, electron mobility analogs to metal, high surface area, and environmentally friendly^14^. The GO has different oxygen-containing functionalities groups including ketone (–C=O), carboxyl (–COOH), carbonyl (–C=O), epoxy (–C–O–C–), and hydroxyl (–OH) groups due to oxidization of natural graphite powder^15,16^. These functional groups have highly strong oxidizing characterization which causes their use in different fields like heterogeneous catalysts^17^. Sing and Ali^18^ used tungstophosphoric acid supported graphene oxide as a heterogeneous catalyst for esterification and transesterification reactions. Pala and coworkers^19^ employed GO as a surfactant for determining the stability and physicochemical properties of Mahua biodiesel blend. However, the separation of nano-particles from the reaction mixture are difficult and are possible using centrifuge or filtration^20^. Safaripour et al.^21^ synthesized barium tin oxide-reduced graphene oxide nano composite for biodiesel production. In order to resolve the issue, magnetic nano particles are used. The magnetic catalyst easy separated from products by an external magnet that prevents catalyst wastage and it is easily recovered and reused, in addition to reduce the cost of separation process^22^. Coating Fe_3_O_4_ magnetic nanoparticles caused a high electromagnetic wave and it could be easily separated from the reaction mixture also to prevent catalyst degradation and improve catalyst activity of GO throughout transesterification reaction NaOH be possible to use to recap GO magnetic nanoparticle^23,24^. Aghabeighi et al.^25^ reported biodiesel production by GO-NiFe_2_O_4_ which lipase Immobilized as nano biocatalysts. Jume et al.^26^ described catalyst activity of strontium-titanium trioxide doped on magnetic GO in the transesterification of acidic waste cooking oil. Nematian et al.^27^ synthesized Lipase immobilized on magnetic GO as a heterogeneous catalyst for biodiesel production, the highest biodiesel yield was 71.19%.

In recent years, the Ultrasonic technique was used for biodiesel production. In this method, by passing a direct ultrasonic frequently through the raw materials, the constraint between triglyceride oil and the water is removed and biodiesel production with high yield and also a transesterification process was possible in a short time^28,29^. Mahdi et al. described synthesis of biodiesel via the Ultrasonic method from *Pistacia atlantica mutica* (PAM) as a primary feed stock oil^30^. Khanian-Najaf-Abadi et al. discussed using NaOH catalyst for biodiesel production through Ultrasonic transesterification reaction^31^.

In the current study, electrolytic transesterification of grease trap waste by a novel and active NaOH@GO-Fe_3_O_4_ catalyst with high stability was reported for the first time. The characterization of the catalyst was investigated by Field Emission Scanning Electron Microscopy (FESEM), Fourier-transform infrared spectroscopy (FTIR), X-ray powder diffraction (XRD), and Brunauer–Emmett–Teller (BET) analysis. The effects of reaction parameters like catalyst weight, methanol to oil molar ratio, time, and ultrasonic frequently were studied on biodiesel yield. Finally, a kinetic study of transesterification was performed under optimum conditions for the estimation of the rate constant and activation energy.

## Materials and methods

### Materials

In this experimental study, to synthesize GO, Sulfuric acid (H_2_SO_4_, 98%), Nitric acid (HNO_3_, 65%), Graphite powder, Hydrochloric acid (HCl, 37%), and Potassium chloride (KClO_3_, 95%) were used and Purchased from Merck company. Iron (III) chloride hexahydrate (FeCl_3_·6H_2_O, 95%), Sodium hydroxide (NaOH, 90%), and Iron(II) chloride tetrahydrate (FeCl_2_·4H_2_O, 95%) were used for preparation of Fe_3_O_4_ and purchased from Merck company. All materials used without any purification process.

### Graphene oxide synthesis

The research involved developing GO using a modified version of the Hummers and Offeman technique, which overcomes past limitations and yields pure and high-quality GO^32^. To create the GO, graphite powder was combined with a blend of nitric acid and sulfuric acid in a 1:2 ratio. The mixture was then chilled in an ice bath and slowly mixed with potassium chlorate. After seven days, the color of the solution changed to green, and prepared sample as the oxidized suspension was purified using hydrochloric acid (5 wt%) to remove impurities, and deionized water several times until pH reached to 7. The final product dehydrated at 60 °C for 12 h in a vacuum oven.

### Synthesis of GO-Fe_3_O_4_ and NaOH@GO-Fe_3_O_4_

**Figure 1:**
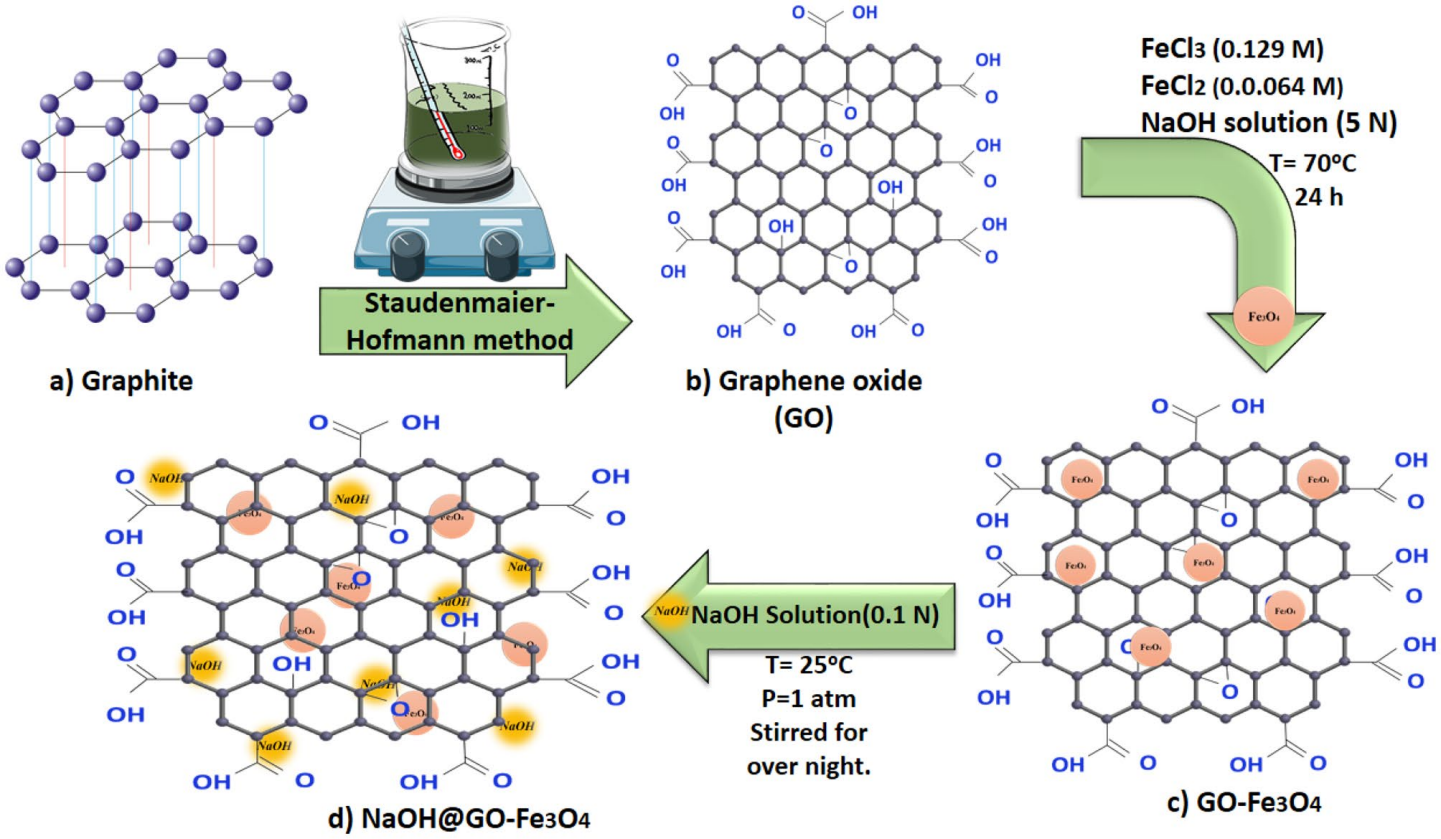
Steps of synthesis magnetic catalyst NaOH@GO-Fe_3_O_4_.

After subjecting 1 g of GO to ultrasonic waves for 30 min, a mixture of FeCl_3_·6H_2_O and FeCl_2_·4H_2_O (2:1 molar) was added and stirred for another 30 min. The addition of NaOH solution caused the color of the mixture to turn black, after which it was stirred for 24 h by a laboratory shaker. An external magnet was used to separate NaOH@GO-Fe_3_O_4_. Finally, prepared sample dried in a vacuum oven at 70 °C. The GO-Fe_3_O_4_ sheets were then crushed into small powder and finally calcined at 300 °C for 3 h^33^. Figure 1 shows steps of synthesis magnetic catalyst.

### Characterization analysis

The raw GO, GO-Fe_3_O_4_ and NaOH@GO-Fe_3_O_4_ as a magnetic catalyst were determined and characterized by various analysis to investigate their morphology, elemental analysis, crystal structure, and surface area. Field Scanning Electron Microscopy (FESEM, Philips XL30 ESEM) was utilized to observe the surface morphologies of the catalyst and conduct elemental analysis. The range of 2θ = 5–80° was used for X-ray Powder Diffraction (XRD) analysis, with a step size of 0.05° (XRD, PHILIPS PW1730). Fourier Transform Infrared spectroscopy (FTIR, Thermo, Avatar) was used to examine molecular vibration characterization of catalysts and oil between 400 and 4000 cm^−1^. The determination of catalyst morphologies and surface area of a mesoporous solid were carried out using BET analyses (BJH, BELSORP MINI II), while a non-porous solid was analyzed for pore size distribution.

### Ultrasonic technique

**Figure 2:**
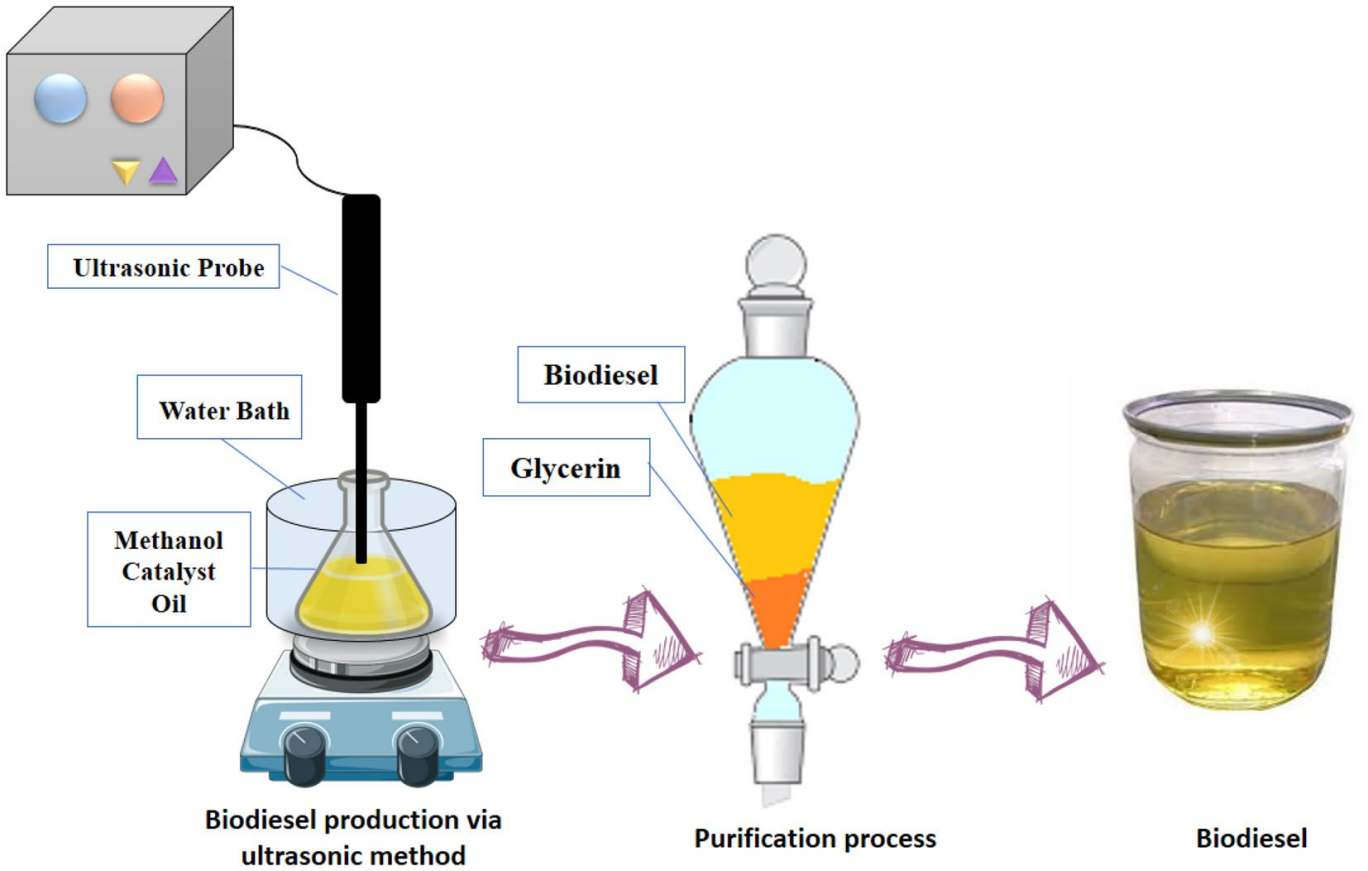
Scheme of biodiesel production via ultrasonic method.

The experiment was conducted using an ultrasonic system with various ultrasonic frequencies (Fig. 2). Concisely, 50 g of waste cooking oil was added in reaction cell, then different amount of catalyst weight (3–5 wt%), methanol to oil molar ratio (5:1 to 20:1) increased to the reaction cell. Finally different ultrasonic frequently (35–55 kHz) and reaction time (15–45 min) were tested to the cell. All experimental runs was done at a constant temperature of 60 °C and mechanical constant rate (500 rpm). At the end, final reaction mixture containing biodiesel as product and glycerol as a by-product were transferred to the separated funnel. Finally, the biodiesel as a final product clean with distillated water several times until neutralization. Then, it heated at 110 °C to remove excess water and methanol. The efficiency of biodiesel calculated with equation below:

1 $$Biodiesel\;yield(\% ) = \left( {\frac{weight\;of\;biodiesel}{{weigh\;of\;oil}}} \right) \times 100$$

### Experimental design

In order to enhance the process of optimization of biodiesel yield, the central composite design (CCD) based on response surface methodology (RSM) was employed. The study focused on three independent variables: catalyst weight (A), methanol to oil molar ratio (B), time (C), and ultrasonic frequently (D), while the dependent variable or response was biodiesel yield. The weight of the catalyst was kept stable with low variations during the transesterification reaction and was not considered as a factor. Table 1 outlines the lower and upper levels for each independent factor and shows their codes. To determine absolute errors, thirty experiments were conducted, with six replication sets at central points, such as eight axial, sixteen factorial points, and six central. The results of these experiments are presented in Table 3.

**Table 1:** Lower and high levels of independent factors Based on BBD.

Factor	Sign	Units	Low	High
methanol to oil	A	molar ratio	5	20
Catalyst weight	B	wt%	3	5
Time	C	min	15	45
Ultrasonic frequently	D	kHz	35	55

2 $$R={\alpha }_{0}+\sum_{i=1}^{k}{\alpha }_{i}{X}_{i}+\sum_{i=1}^{k}{\alpha }_{ii}{X}_{i}^{2}+\sum_{i=1}^{2}\sum_{j=i+1}^{3}{\alpha }_{ij}{X}_{i}{X}_{j}+\varepsilon$$

The yield of biodiesel, denoted as Y, is established through regression programming by computing. These coefficients pertain to the constant (α_0_), interaction factors (α_ij_), quadratic (β_ii_), and linear (α_i_), respectively. X_i_ and X_j_ are considered independent variables while ε denotes the error. The adjusted R-squared (R^2^_adj_) and R squared (R^2^) were calculated by Eqs. (3) and (4), respectively^34^.

3 $$R_{adj}^{2} = 1 - \frac{{SS_{Residual} /DF_{Residual} }}{{(SS_{\bmod el} + SS_{Residual} )/(DF_{\bmod el} + DF_{Residual} )}}$$

4 $$R^{2} = \frac{{SS_{Residual} }}{{SS_{model} + SS_{{{\text{Re}} sidual}} }}$$

where *SS value* is the sum of squared and the amount of *DF* is the degree of freedom.

### Kinetic study

A kinetic model can be utilized to determine the rate constant and activation energy for the production of biodiesel from waste cooking oil. The study kept the reaction parameters constant, including catalyst weight, methanol to oil molar ratio, time, and ultrasonic frequency, but varied the temperatures (45, 55, and 65 °C). Biodiesel yield was measured by online product sampling at specific intervals. The lowest temperature of 45 °C was chosen due to the sluggish production of glycerol, while the highest temperature of 65 °C was selected as it was near the boiling point of methanol^35^. The first-order rate equation show in the Eq. (5).

5 $$Rate = - r = - \frac{d[TG]}{{dt}} = K[TG]$$

TG represents the triglyceride’s concentration, K shows the constant rate (min^−1^), and t represents the time taken for the reaction. The following equation can be used to measure Eq. (5):

6 $$- \ln \left( {\frac{TG}{{TG_{0} }}} \right) = kt$$

where *TG*_*0*_ and TG are the primary concentration of triglyceride (*%*) and the terminal concentration (*%*), respectively. the conversation of factor *X* was showed by Eq. (7):

7 $$X = \frac{{[TG]_{0} - [TG]_{t} }}{{[TG]_{0} }}$$

The *K* (min^−1^) for the transesterification in each of temperatures can be calculated by \the data got from the Eq. (8):

8 $$- \ln (1 - X) = {\text{Kt}}$$

The *Arrhenius* equation (Eq. (9)) shows the energy of activation, applying information of constant speed and temperature according to the data got from the kinetic experimental.

9 $$K = Ae^{{\frac{{ - E_{a} }}{RT}}}$$

where *K* and *A* are the velocity constant and *Arrhenius* constant, respectively. The *E*_*a*_ is the activation energy (J/mol)*, R* is the universal gas constant (J/mol/K) and *T* is absolute temperature (K)^36^.

### Properties of biodiesel

The physiochemical characterization of biodiesel from waste cooking oil like cloud point (ASTM D 2500), flash point (ASTM D 93), pour point (ASTM D 97), kinematic viscosity at 40 °C (ASTM D 445), density at 15 °C (ASTM D 1298), iodine value (ASTM D 874), cetane number, saponification value (ASTM D 97) were determined by the method of ASTM standards.

## Result and discussion

### Study of and structural, thermal properties of magnetic catalyst

#### Morphological and elemental analysis

**Figure 3:**
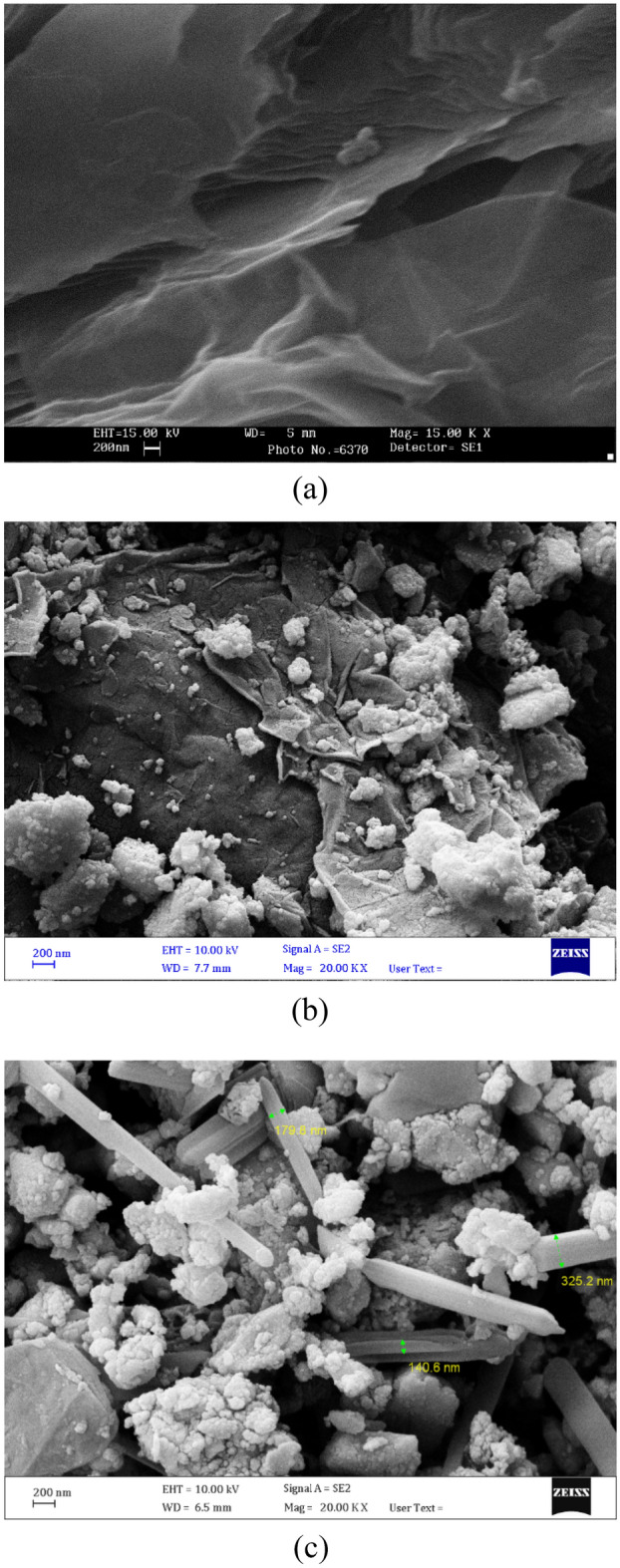
FESEM images of (**a**) raw GO, (**b**) GO-Fe_3_O_4_ (**c**) NaOH@GO-Fe_3_O_4_.

Morphological structure of pure GO, GO-Fe_3_O_4_, and NaOH@GO-Fe_3_O_4_ as magnetic heterogeneous catalyst after immobilizing NaOH have showed in Fig. 3. According to the FESEM images for pure GO, the morphological of GO layers were ruffled and twisted across texture. Shemshani et al.^37^ believed that a functional oxygenated groups cause these structure. In addition, the thin and flexible layers of raw GO are easily seen. In Fig. 3b, the spherical shape of Fe_3_O_4_ nanoparticles was observed on GO sheets. As well as they are uniformly scattered and twisted to both the inner and surface of GO layers. These results have good understanding with another reports^38,39^. It can be clearly seen that in Fig. 3c, the natural magnetic properties of Fe_3_O_4_ nanoparticles have spherical-shaped of exists as agglomerates. In addition, after calcination of NaOH, they have needle-shaped crystals on the surface and inner of GO sheets.

#### Determining crystal structural

**Figure 4:**
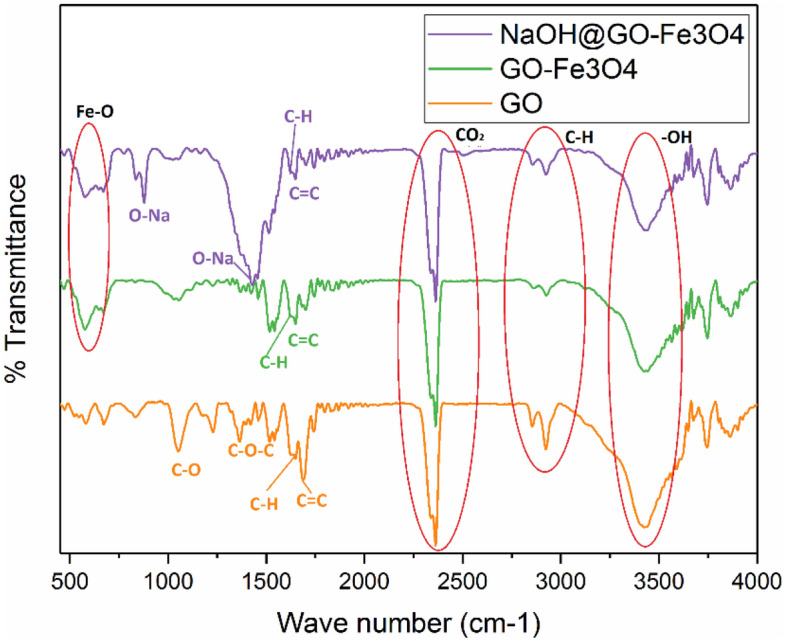
FTIR spectrum of (**a**) raw GO, (**b**) GO-Fe_3_O_4_as magneticcatalyst support (c NaOH@GO-Fe_3_O_4_.

In order to study molecular vibration of various functional groups of pure GO, GO-Fe_3_O_4_, and NaOH@GO-Fe_3_O_4_ FTIR analysis was used and showed in Fig. 4. The epoxy (C–O) group of pure GO has main peak at 1044 cm^−1^ and C–O–C groups has a main peak at and 1415 cm^−1^, respectively. The C=C bands in aromatic groups have a peak at 1624 cm^−1^. The C=O carboxyl groups on GO layers have a peak at 1721 cm^−1^. The board and main peak which observes at 3431 cm^−1^ related to the stretch and binding vibration of –OH groups. FTIR analysis confirmed that GO layers have different special functional groups on their inner layer and surface, these functional groups assist to create binding sites with other materials like Fe^40^. These results are similar to the FTIR spectra of GO which are reported by other researchers^15,16^.

Although all peaks of raw GO are observed in the FTIR spectrum of GO-Fe_3_O_4_ which indicated after immobilization Fe, the structure of GO does not change, however, chemical deposition of Fe_3_O_4_ nano particles on the GO layers declines severity of the peaks. The GO-Fe_3_O_4_ showed following main peaks: the carbonyl, C=C aromatic groups, and –OH groups have main peaks at 1415 cm^−1^, 1621 cm^−1^, and 3424 cm^−1^, respectively. The peak at 1060 cm^−1^ which related to the C-O stretching vibration of epoxy groups. The specific and sharp peak at 567 cm^−1^ assigned to Fe–O with the stretching vibration. Based to the FTIR analysis results, the GO surface was improved by Fe_3_O_4_ and the experimental results was similar to other reports^41,42^.

The same peaks GO-Fe_3_O_4_ peaks with a light difference are observed in the FTIR spectra of NaOH@GO-Fe_3_O_4_. The FTIR spectra of magnetic catalyst possess the peaks at 3426 cm^−1^, 1636 cm^−1^, 1365 cm^−1^, and 1052 cm^−1^ which related to the –OH, C=C, carbonyl, and C–O groups, of pure GO, respectively. As well as, the specific peak observed at 575 cm^−1^ related to the Fe–O groups. In FTIR spectra of magnetic catalyst, the maiden peak was appeared at 1649 cm^−1^ corresponded to the –OH groups. The peaks which appeared at 1429 cm^−1^ and 877 cm^−1^ can be related to the O-Na deformation and plan bending of O-Na^43^. The peak at 575 cm^−1^ associated to the Fe–O–Fe in Fe_3_O_4_. According to the FTIR analysis results, after immobilization of NaOH, although the morphological and surface of GO-Fe_3_O_4_ as magnetic catalyst support was changed, NaOH@GO-Fe_3_O_4_ magnetic catalyst was successfully generated.

**Figure 5:**
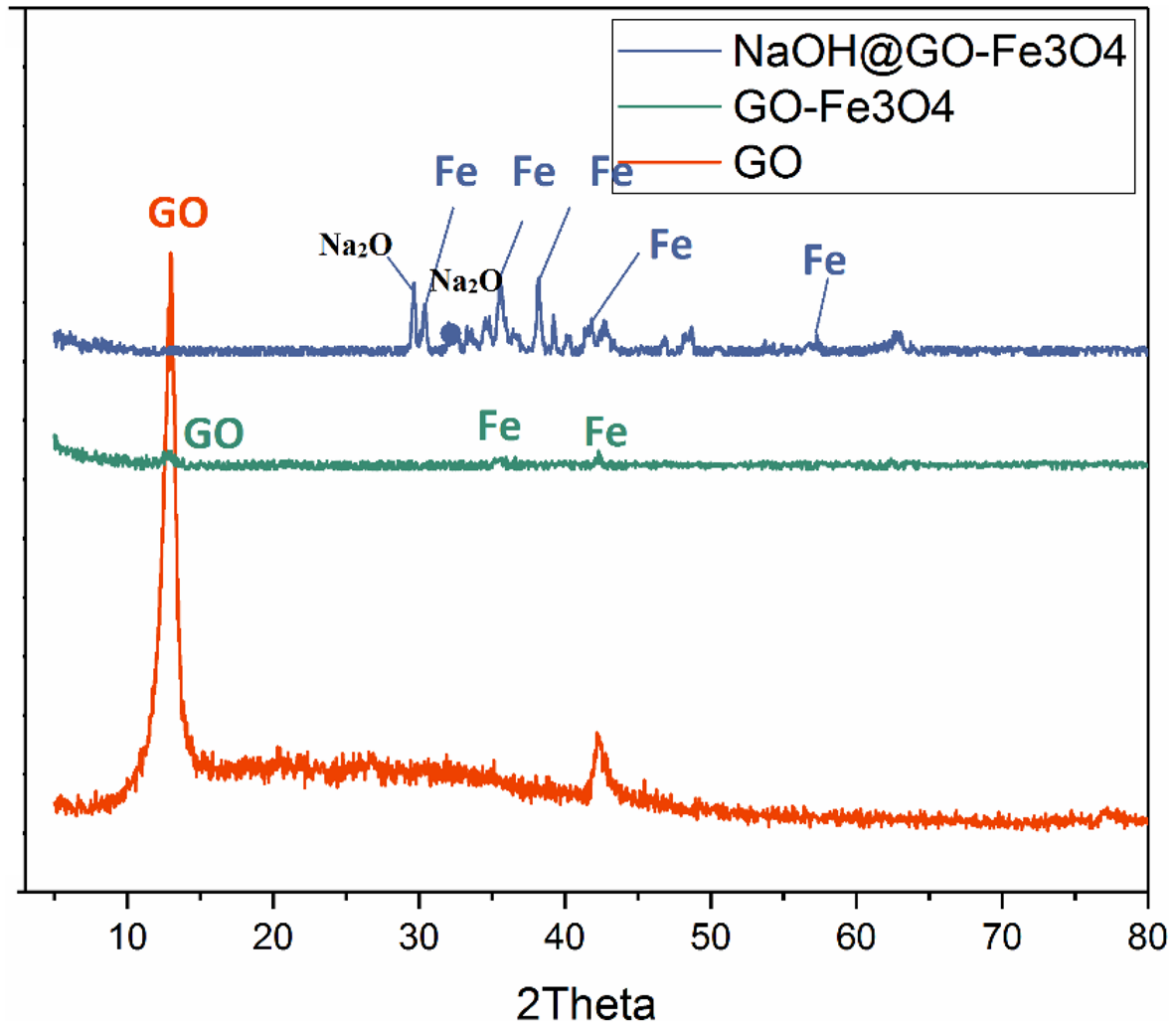
XRD diffraction patterns of (**a**) raw GO, (**b**) GO-Fe_3_O_4_as magnetic catalyst support (**c**) NaOH@GO-Fe_3_O_4_.

The crystal structure of pure GO, GO-Fe_3_O, and NaOH@GO-Fe_3_O_4_ (Fig. 5) was studied by the XRD analysis. The single sharp peak clearly seen at 2θ = 12.8° with d-spacing of 7.02 Å corresponded to the crystal face of GO^44^. The characteristic peaks of GO-Fe_3_O_4_ with low intensity were appeared at 30.72°, 35.6°, 42.44°, 57.28°, and 61.6° which showed Fe_3_O_4_ magnetic nano particles with the cubic spinel crystal phase (JCPDS Card. PDF No. 85-1436)^20^. While the severity of GO peak at 2θ = 12.5° was decline significantly. This is because after the coating of Fe_3_O_4_ nano particles, GO sheets hardly stack on together to prepare crystalline structure^45^. All in all, the board small peak illustrated that the synthesis of GO-Fe_3_O_4_ was successfully. The GO-Fe_3_O_4_ have similar peaks with NaOH@GO-Fe_3_O_4_. But some peaks were observed at 18.2°, 30.0°, 33.36°, 35.52°, 42.80°, 53.72°, 57.28°, 63° which were related to the NaOH@GO-Fe_3_O_4_. Deng et al.^46^ announced that the peaks at 2θ = 31.0°, 35.52°, 42.80°, and 57.28° were assigned to the maghemite or magnetite, the peaks at 2θ = 52.5° and 63° were associated to the hematite, and the magnetic composite structure has peaks at 2θ = 19.2° and 34.36°. The new peak observed at 2θ value of 33.36° was related to the Na_2_O phase as well^47^. Calcination process of catalyst causes that the NaOH molecules converted to the Na_2_O, therefore, magnetic catalyst have high alkaline characterization. In order to measure the particles size of magnetic catalyst the Debye–Scherrer equation (Eq. 10) was used:

10 $$S = 0.98\lambda /\beta \cos \theta$$

where *S* is the size of crystalline. λ , β and θ are x-ray wavelength line, width at half highest of the peaks of a radian and *Bragg* angle, respectively. The nanoparticles size of NaOH@GO-Fe_3_O_4_ is an average size 14.2\2 nm.

#### Investigating surface area and pore volume

The specific surface area (S_BET_), total porosity of particles (*V*_*p*_), average pore diameter (nm) of NaOH@GO-Fe_3_O_4_ magnetic catalyst were studied by BET analysis. The results of BET analysis are displayed in Table 2. The general surface area of solid catalyst was 20.07 m^2^/g. the mean pore diameter and general porosity of particles were obtained 14.05 nm and 15.06 cm^3^/g, respectively. Based on the IUPAC categorize, NaOH@GO-Fe_3_O_4_ has mesoporous pores^48^.

**Table 2:** BET analysis results for NaOH@GO-Fe_3_O_4_.

Sample	S_BET_(m^2^/g)	V_total_ (cm^3^/g)	Mean pore diameter (nm)
KOH@GO-Fe_3_O_4_	20.07	15.06	14.05

All in all, WCO as a basic feedstock for biodiesel may be a low-cost and plenteous resource that can diminish the cost of the biodiesel production process. This feedstock, also, is not in the human chain. It can keep up a key separate the competition for arable arrive and water resources that are related to consumable oils as well^49^. It can diminish the characteristic influence of misuse exchange and the outpouring of green gasses, as the carbon contained in misused cooking oil is to an awesome degree biogenic and renewable^50^. NaOH@graphene oxide-Fe_3_O_4_ may well be an attractive magnetic heterogeneous catalyst that can be successfully separated by magnet, keeping up a vital separate from the issues of catalyst loss, wastewater generation, and soap formation that are common with homogeneous catalysts It incorporates a catalytic development and selectivity, as the NaOH gives the fundamental regions for the transesterification, though the graphene oxide and Fe_3_O_4_ overhaul the consistent quality, diffusing, and alluring properties of the catalyst. It can work underneath smooth reaction conditions, such as mild reaction temperature, low catalyst weight, and low oil-to-methanol molar ratio, diminishing essentialness utilization and advancing the biodiesel resign.^51^.

### Response surface methodology (RSM) analysis

The aim of the study was to assess the impact of varying the independent factors of the molar ratio between methanol and oil (A), catalyst weight (B), reaction time (C), and ultrasound frequency (D) on biodiesel yield (%). Using the central composite design method, thirty experiments were conducted to investigate the value of independent parameters and their interactions on biodiesel yield, as outlined in Table 3 upon evaluating various models, operational parameters have quadratic and significant model, with the best regression model expressed in Eq. (3) based on the experimental data. The coefficient of determination (R^2^) was used to determine the state of the quadratic model. The value of R^2^ was 0.9927, predicted-R^2^ was 0.9859, and the Adjusted-R^2^ value was 0.9620. The high value of R^2^ proves that the model could measure the biodiesel yield verified by the mathematical equation. A good agreement was seen between the experimental and predicted biodiesel yield as well.

**Table 3:** RSM based CCD for four independent factors.

Run	A:Methanol to oil molar ratio	B:Catalyst weight wt%	C:Time min	D:Ultrasonic frequently KHz	Biodiesel yield %
1	5	3	45	70	83
2	12.5	6	30	50	57
3	12.5	4	30	10	81
4	12.5	2	30	50	73.5
5	5	3	15	30	65.5
6	12.5	4	30	50	94.8
7	5	3	45	30	67
8	12.5	4	30	50	95.9
9	20	5	15	70	34
10	20	5	45	70	40
11	12.5	4	30	50	93.2
12	5	3	15	70	65
13	20	5	15	30	59
14	12.5	4	30	50	95
15	12.5	4	30	50	92.8
16	12.5	4	30	90	73
17	12.5	4	60	50	62
18	12.5	4	0	50	46
19	5	5	45	30	86
20	20	3	15	30	70.5
21	20	3	45	70	69
22	20	5	45	30	53
23	5	5	15	70	42
24	27.5	4	30	50	33
25	20	3	45	30	53.12
26	12.5	4	30	50	92.6
27	20	3	15	70	68
28	2.5	4	30	50	56
29	5	5	15	30	65
30	5	5	45	70	73

10 $$Y=94.35-6.08\times A-5.09\times B+3.63\times C-2.55\times D-3.76\times AB-5.49\times AC-3.06\times BC-6.43\times BD+3.56\times CD-12.07\times {A}^{2}-6.89\times {B}^{2}-9.70\times {C}^{2}-3.95\times {D}^{2}$$

The objective of this study was to evaluate the influence of various independent variables on biodiesel yield (%), including A, B, C, D. The central composite design method was used to perform 30 experiments and assess the individual variables and their interactions on biodiesel yield (Table 3). The Analysis of variance (ANOVA) was used to calculate interaction between independent parameters such as catalyst weight (3–5 wt%), methanol to oil molar ratio (5:1 to 20:1), ultrasonic frequency (35–55 kHz), and reaction time (15–45 min) (Table 4), and determine the optimal conditions. Variables with higher F-values and smaller P-values has a necessary effect on biodiesel production as a response, while AD did not. The insignificant lack of fit further confirmed the model's adequacy, and the coefficient of variance (CV) demonstrated its high accuracy.

**Table 4:** ANOVA for Quadratic model.

Source	Sum of Squares	df	Mean Square	F-value	p-value	significant
Model	9991.63	14	713.69	145.88	< 0.0001	*
A-Methanol to oil	886.71	1	886.71	181.24	< 0.0001	*
B-Catalyst weight	621.39	1	621.39	127.01	< 0.0001	*
C-Time	316.25	1	316.25	64.64	< 0.0001	*
D-Ultrasonic frequently	155.65	1	155.65	31.82	< 0.0001	*
AB	225.90	1	225.90	46.17	< 0.0001	*
AC	482.68	1	482.68	98.66	< 0.0001	*
AD	1.06	1	1.06	0.2168	0.6481	-
BC	149.33	1	149.33	30.52	< 0.0001	*
BD	661.52	1	661.52	135.21	< 0.0001	*
CD	202.21	1	202.21	41.33	< 0.0001	*
A^2^	3998.41	1	3998.41	817.27	< 0.0001	*
B^2^	1300.67	1	1300.67	265.86	< 0.0001	*
C^2^	2580.09	1	2580.09	527.37	< 0.0001	*
D^2^	427.68	1	427.68	87.42	< 0.0001	*
Residual	73.39	15	4.89			*
Lack of fit	64.11	10	6.41	3.46	0.0917	-
Pure error	9.28	5	1.86			
Cor total	10,065.02	29				

**Figure 6:**
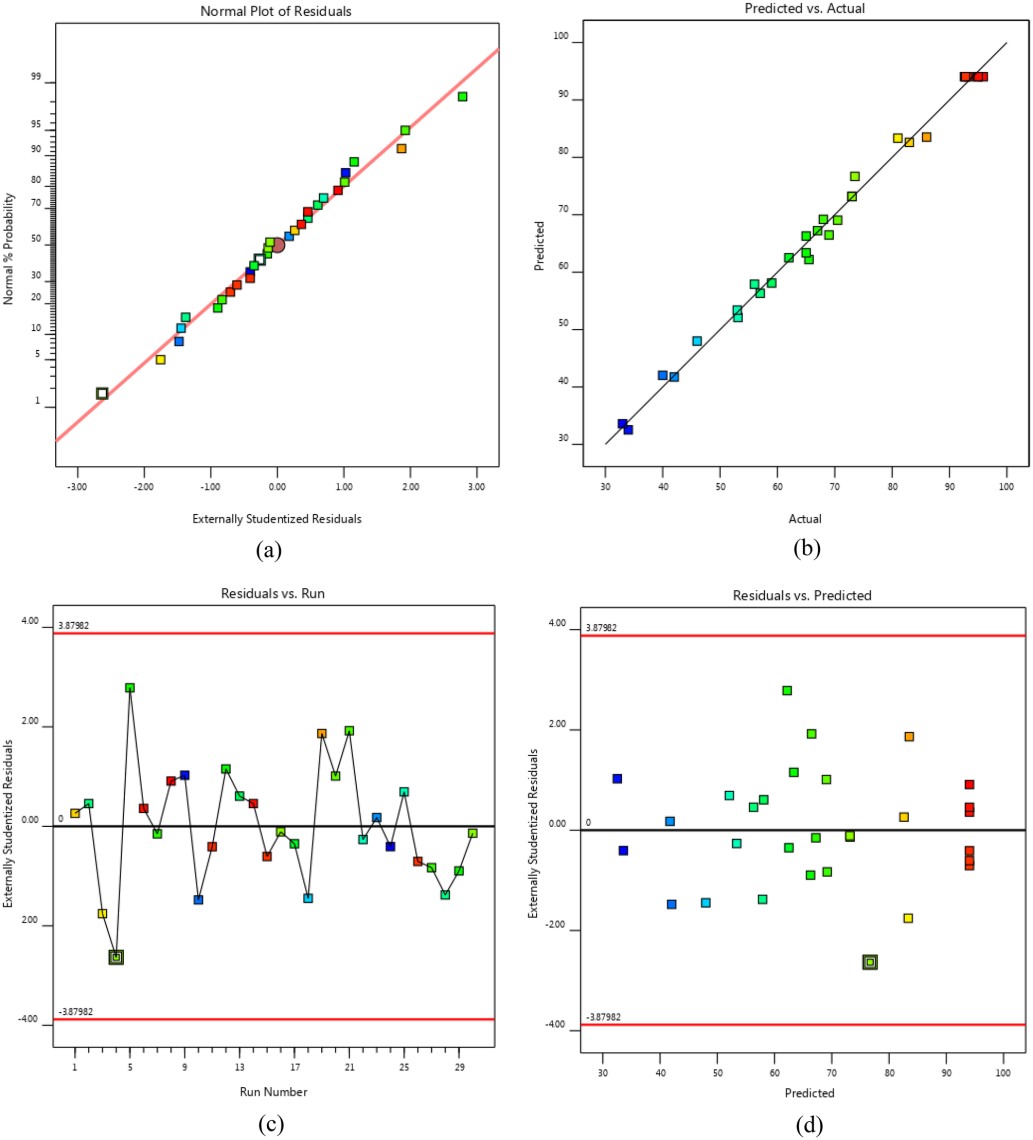
(**a**) Actual versus predicted data (**b**) Normal probability plot of residuals. (**c**) Outlier t plot (**d**) Residual plot.

Figure 6a displays both the predicted and actual Biodiesel yield values, while Fig. 6b shows a normal probability plot indicating that the data is normally distributed^48^. If the curve follows an S-shape, it is incorrect to use the model, and an additional response transformation is necessary [48). The outlier t plot for all response runs is presented in Fig. 6c, which identifies runs with high residuals. The majority of residuals should fall within + 3.87982 and -3.87982 that indicate two types of error including operational and positional errors in the empirical data and mathematical model, respectively. As no data points fall outside this range, the results suggest that all data are compatible with this model. Figure 6d displays the studentized residuals against predicted response, which should be randomly distributed to demonstrate that changes in primary observations not relevant to the amount of response. All data do not use particular patterns for scattering in Fig. 6d supports the suggested model as a precise representation of the transesterification process.

#### Effects of operating independent factors on transesterification reaction

**Figure 7:**
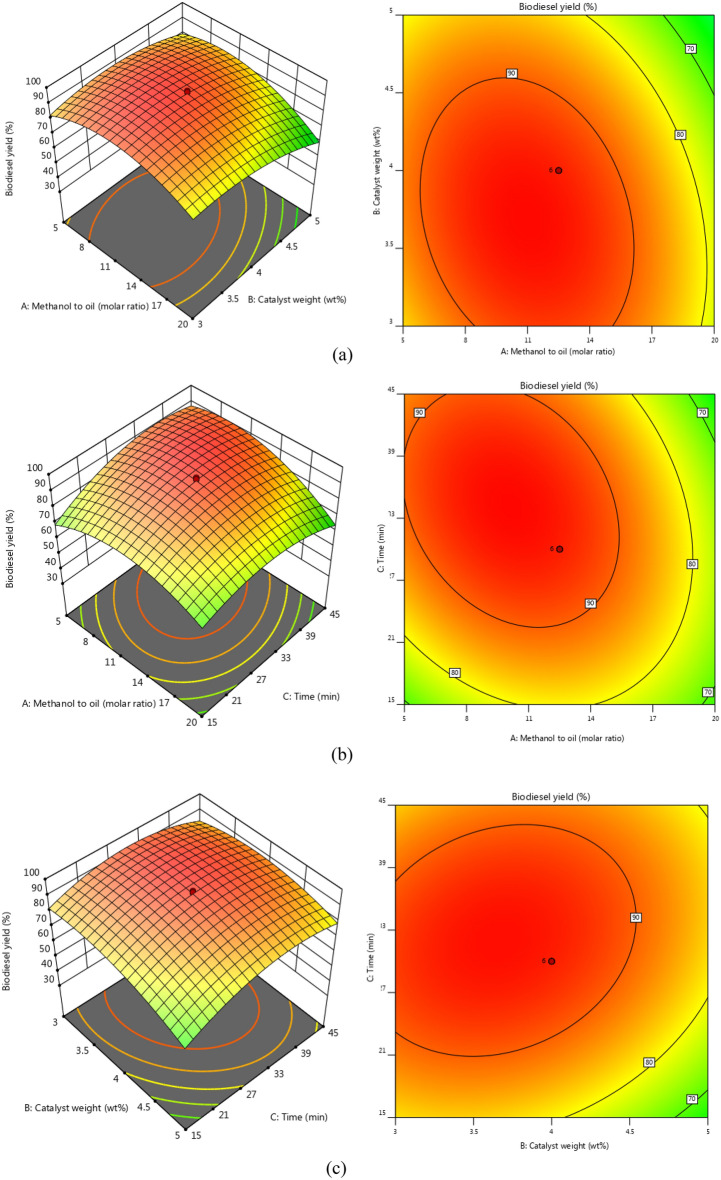

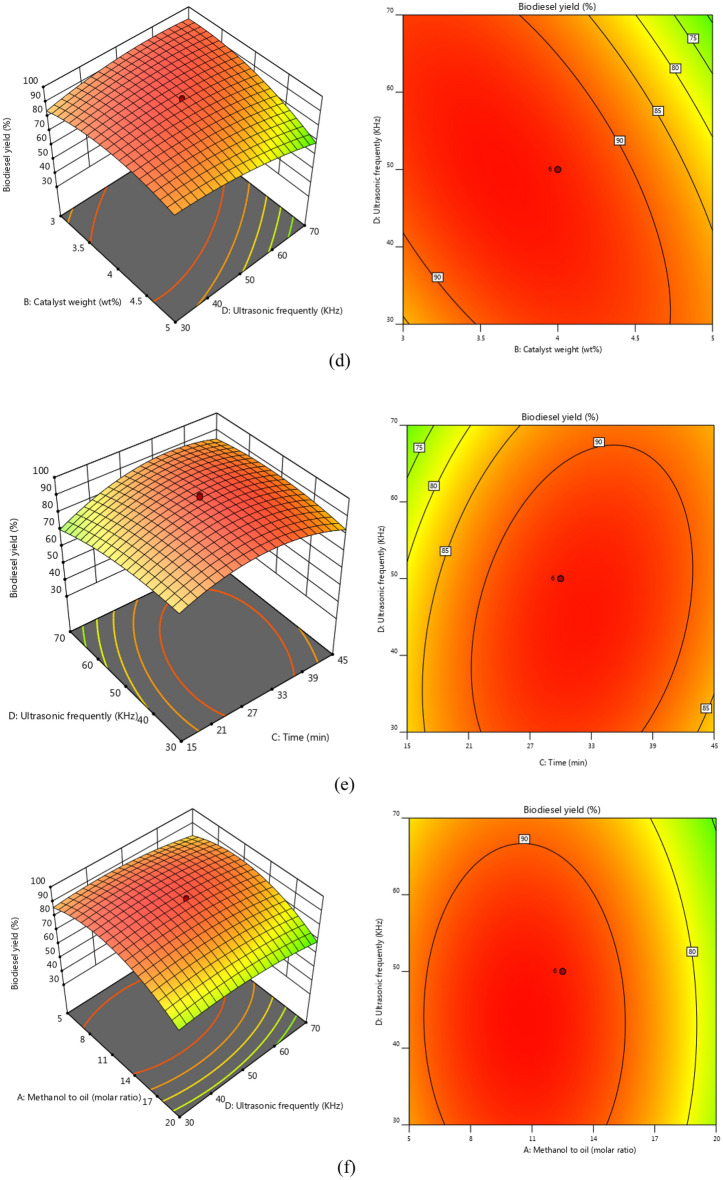
The 3D surface and 2D contour plots of interaction between (**a**) catalyst weight and methanol to oil molar ratio, (**b**) reaction time and methanol to oil, (**c**) catalyst weight and reaction time, (**d**) ultrasonic frequency and catalyst weight, (**e**) ultrasonic frequency and reaction time, (**f**) Ultrasonic frequently and methanol to oil.

The biodiesel yield is impacted by two independent variables, with Fig. 9 displaying 3D and 2D plots. The methanol oil molar ratio serves as the primary factor influencing the biodiesel yield. As demonstrated in Fig. 7a,b,f, an enhancing in this independent factor causes that biodiesel yield increases, with a maximum of 95.88% achieved at a ratio of 10.52:1. However, by enhancing methanol to oil molar ratio higher than optimum condition lead to biodiesel yield decreases^52^

The catalyst weight (B) is a primary factor in transesterification reaction. The value of the weight of NaOH@GO-Fe_3_O_4_ was changed from 3 to 5wt%. Based on the results of Fig. 7a,c,d. The NaOH@GO-Fe_3_O_4_ considers as an active catalyst due to high alkali sites that have positive effect on the interaction between triglyceride oil molecules and methanol molecules that cause biodiesel produced with highest yield [71). Therefore, only 3.76 g of alkali magnetic catalyst could generated biodiesel with high yield of 95.88%. However, increasing catalyst more than 3. 76 g has a negative effect on the transesterification reaction due to mass transfer resistance, All in all, when the final biodiesel yield decreases, the reaction mixture was viscose, and the mixture agents harder^47^.

The results presented in Fig. 7b,c,e demonstrate that the maximum response was obtained after a certain period of time was obtained after a certain period of time. Increasing the reaction time from 5 to 33.29 min resulted in an increasing biodiesel yield to 95.88%. During the initial stages of the reaction, methanol reacted with the oil molecules at a slow rate. However, after 33.29 min, the reaction rate improved and more biodiesel generated because the triglyceride oil molecules have sufficient time to participate in the transesterification reaction. The transesterification rate enhanced due to the molecules reacting together. The biodiesel yield, however, declined after the optimum reaction time due to ester hydrolysis and the reversible reaction of transesterification. Therefore, the optimum reaction time was chosen at 33.29 min.

Figure 7d–f shows the Ultrasonic frequently impacts multiple from 35 to 55 kHz. Biodiesel yield increased to 95.88% at the Ultrasonic frequently of 49.58 kHz. Increasing Ultrasound frequency can raise the adsorption rate and indeed, the rate of CH_3_O^−^ ions generation. Convection that was produced by ultrasound assists in the scattering of the organic phase and aqueous phase into each other. Therefore, the formation of an emulsion causes a high interfacial area. In addition, cavitation bubbles provide convection via the production of acoustic waves that generate emulsion of the two phases. Although the magnitude of these waves declines significantly at higher temperatures, it could be attended that the level of the convection level in the medium should decrease with temperature^53^ so 49.58 kHz was selected as the optimum ultra-frequency.

### Optimization of biodiesel production

**Figure 8:**
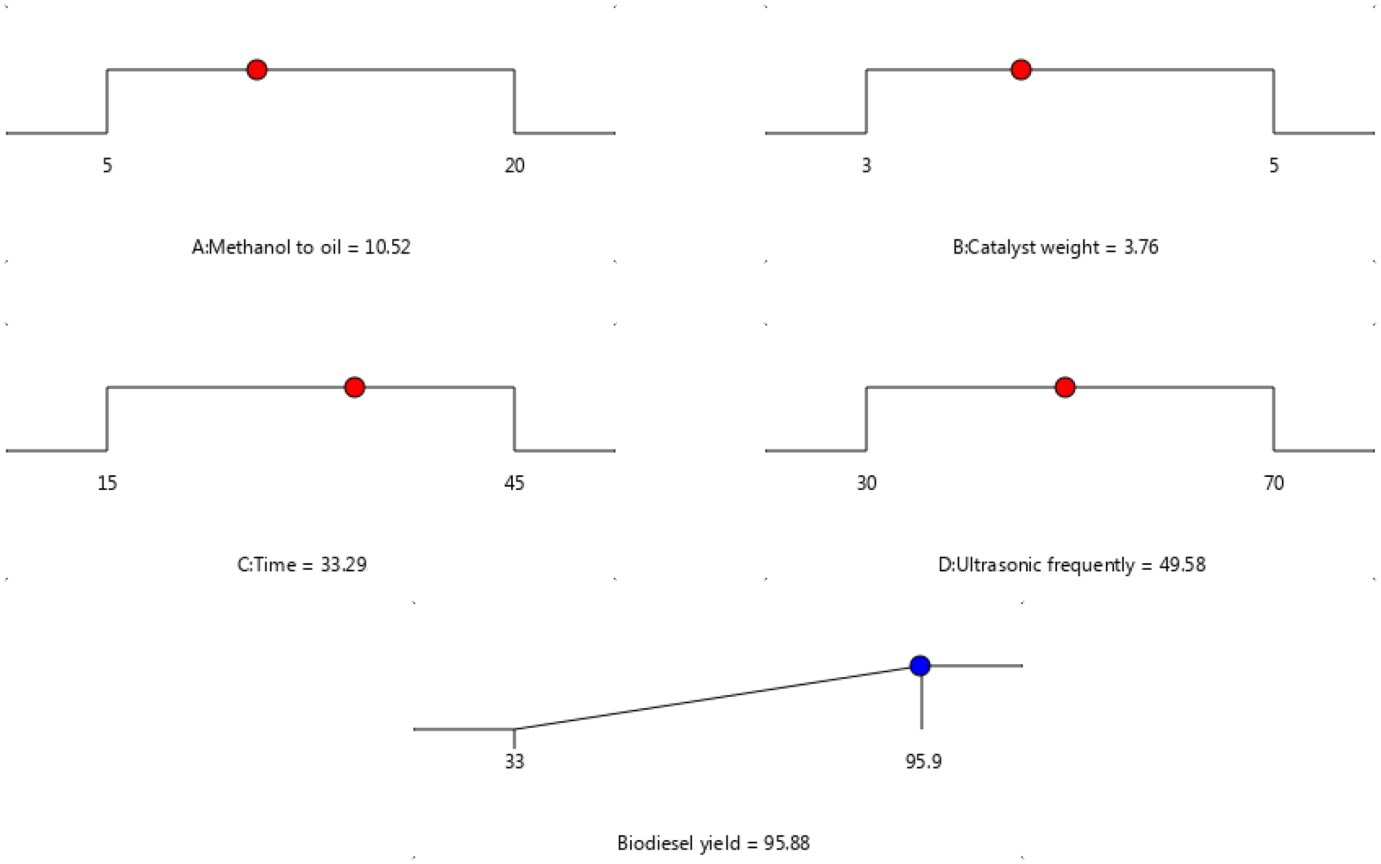
Reaction condition of transesterification reaction under optimum condition.

The RSM-CCD with four independent factors was utilized to optimize the factors within the determined range, taking into account the standard error (StdErr) present in the numerical model. Figure 8 illustrates the optimal values of each factors among the highest and lowest limits. The second-order quadratic polynomial model was employed to determine both the highest biodiesel yield and the optimum factors, which was predicted to be 95.88%. The optimal conditions for achieving this yield were a 10.52:1 molar ratio of methanol to oil, a catalyst weight of 3.76 wt%, a voltage of 49.58 kHz, and a time of 33.29 min. However, the actual yield obtained was very close to the predicted value.

### Characterization of biodiesel

The characteristics of fuel are highly influential in determining how the addition of biodiesel affects diesel engine performance, combustion, and emissions. Table 5 outlines key parameters, including pour point, cloud point, flash point, ash content, Saponification Value, Iodine value, acid value, viscosity, density, and cetane number. Viscosity and density are particularly important as they impact on efficiency of both combustion of fuel, and fuel injection. The amount of saponification indicates the acyl group quantity in the oil phase, while the position of unsaturation in oil was measured by iodine value. The cetane number is a crucial parameter that gauges combustion lag cycle in diesel engines. The ideal biodiesel should meet ASTM standards and exhibit a high flash point, in addition, high cloud point and low pour point perform optimally in various climate conditions.

**Table 5:** Physicochemical properties of ASTM D6751 and biodiesel.

Index	Units	ASTM D6751	Biodiesel
Pour point	°C	− 15 to 10	− 6.369
Cloud point	°C	− 3 to 12	3.428
Flash point	°C	Min 130	171
Viscosity (40 °C)	mm^2^/s	1.9–6	3.650
Density (15 °C)	g/cm^3^	0.881	0.872
Saponification value	mg KOH/g oil	< 500	224.895
Iodine value	gI_2_/g	< 120	119.2
Cetane number	–	> 47	45.216

### Kinetic study

**Figure 9:**
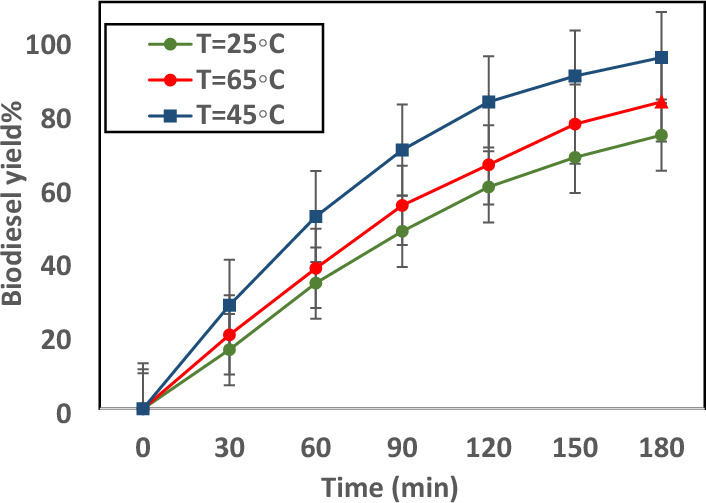
Plot of biodiesel yield as response versus reaction time at different temperatures.

**Figure 10:**
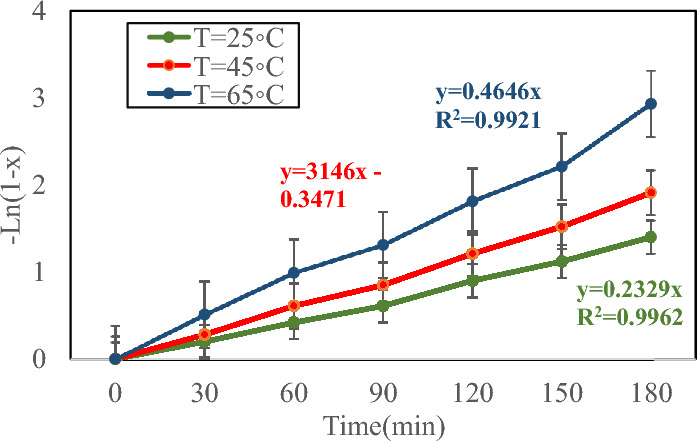
Plot of − *Ln*(1 − *X*) versus reaction time at different temperatures.

Figure 9 illustrates the biodiesel yield (%) as a response against reaction time at various temperatures from 25 to 65. Clearly, the transesterification rate was gradual at the first step of the process, but the rate intensely enhanced with passing time. Therefore, biodiesel yield is increased linearly^54^. For a stable reaction rate module at various temperatures (25 °C, 45 °C, and 65 °C), the plot between − Ln(1 − X) versus reaction time displays the biodiesel yield as can be seen in Fig. 10. A linear equation is observed in all temperatures. Therefore, it concluded that biodiesel production is a pseudo-first-order reaction. For all curves, the *R*^*2*^ values (25 °C—0.2329, 45 °C—0.3146, and 65 °C—0.4646) were more than 0.9 which proved the high accuracy of the porposed correlations. The activation energy of process is 49.2 kJ/min and Arrhenius constant of transesterification is 16.47 * 10^10^ min^−1^.

### Catalyst regeneration

**Figure 11:**
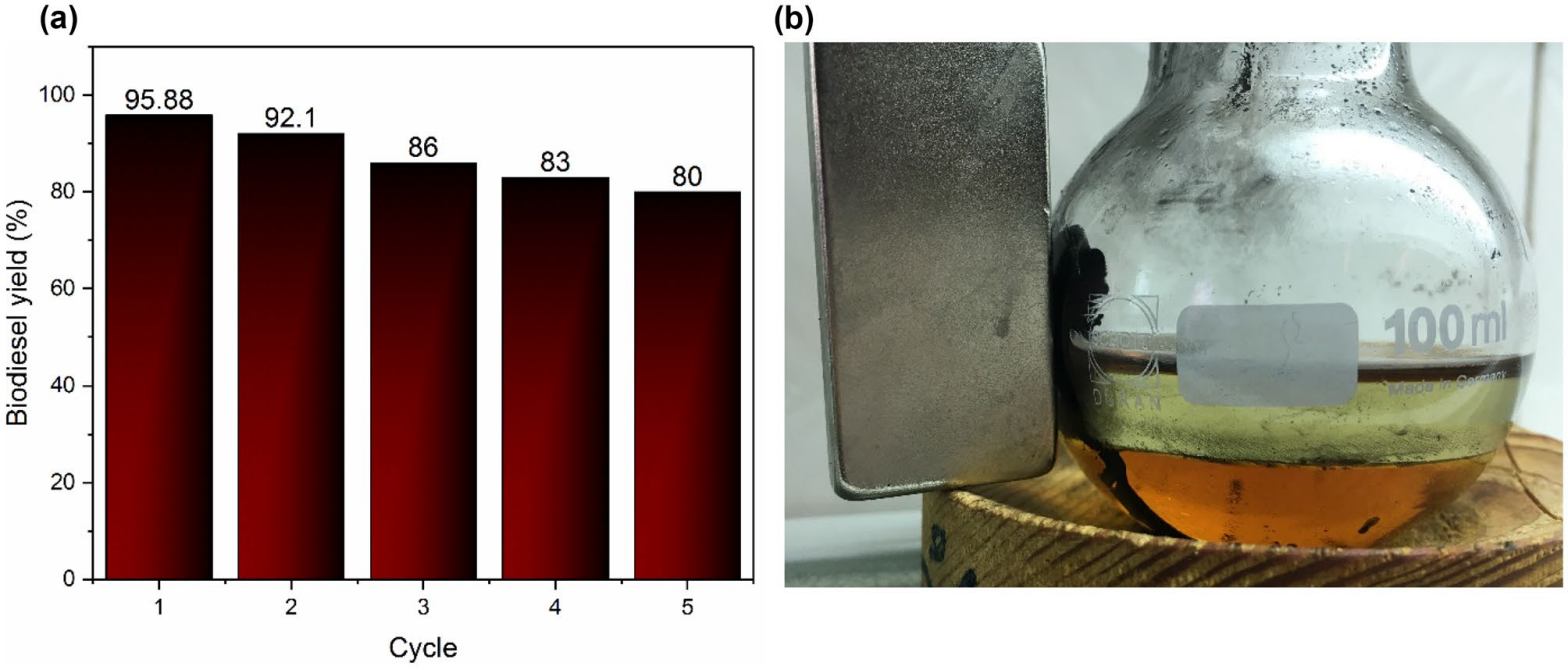
(**a**) Reusability of catalyst, (**b**) NaOH@GO-Fe_3_O_4_ as a magnetic catalyst was separated by magnet from reaction mixture.

Regeneration catalyst is a significant factor in the transesterification reaction, catalyst should be recycled as many times without significant function (Fig. 11). At the end of the reaction, magnetic catalyst was separated by magnet, then washed with n-hexane in order to remove impurities, next dried at oven over night, finally used for biodiesel production. Based to the regeneration process, NaOH@GO-Fe_3_O_4_ as a magnetic catalyst reused five times without decline significantly. Tan et al.^55^ reported that catalyst surface was recovered by larger molecular oil, therefore active sites deactivate and biodiesel yield declined.

### Comparison of results with other studied

The optimum reaction conditions and final biodiesel yield of this study and other reports that used magnetic GO as a base of heterogeneous catalysts were summarized in the Table 6. From the comparison, it is concluded that the NaOH@GO-Fe_3_O_4_ as a magnetic catalyst could generate biodiesel in a short time (33.29 min). That is because the ultrasonic method is a technique that uses high-frequency sound waves to create bubbles in the liquid mixture of oil and alcohol. These bubbles collapse violently, generating intense heat and pressure that accelerate the transesterification reaction. This process enhances the mass transfer and mixing of the immiscible reagents, reduces the catalyst loading and reaction temperature, and increases the biodiesel yield and conversion rate. Therefore, the consumption of energy significantly decreased. By using only 3.76 wt% magnetic catalyst, biodiesel was produced with a high yield (95.88%) as well. The consumption of methanol to oil was 10:1 molar ratio so low reactive materials such as methanol and catalyst were used. At the end of each cycle, the magnetic catalyst was separated by an external magnet, therefore, the cost of using a centrifuge or anything was eliminated which had a significant effect on the final cost of biodiesel as a product. By considering that NaOH@GO-Fe_3_O_4_ was able to be reused five times, this catalyst has economic viability and is able to be used on a large scale.

**Table 6:** Comparison of optimum reaction conditions and biodiesel yield in literature.

Catalyst name	Catalyst weight (wt%)	Methanol to oil molar ratio	Time (min)	Biodiesel yield (%)	Refs.
ZrO_2_-TiO_2_@MGO	4.75	2.33:1	3	99.32	^56^
titanium oxide-GO-Fe3O4	4	5:1	2400	92	^57^
MGO@TiO_2_Ag	4.15	2.52:1	2.5	96.54	^58^
MnFe_2_O_4_@biochar	1.75	11.34:1	56.12	97.26	^59^
This study	3.76	10.52:1	33.29	95.88	–

## Conclusion

In this research, the optimization of the biodiesel production from WCO using the NaOH@GO-Fe_3_O_4_ as magnetic catalyst was performed via the RSM-CCD. The NaOH@GO-Fe_3_O_4_ has an alkali natural because NaOH changes the GO structures, when Na^+^ ions immobilized with GO structure and strong chemical bonds was created by oxygenated functional groups of GO structure. The highest biodiesel yield was 95.88% subject the optimum conditions of 3.76 wt% NaOH@GO-Fe_3_O_4_, methanol to oil molar ratio of 10.52:1, the reaction time of 33.29 min, and ultrasonic frequency of 49.58 kHz. The actual experimental results showed NaOH@GO-Fe_3_O_4_ showed high activity and stability in biodiesel production. As R^2^, pre-R^2^, and adj-R^2^ had high value, it proved than the proposed mathematical model by RSM-CCD possessed a high accuracy to evaluate the biodiesel yield as a process response According to the kinetic results, the activation energy of the process was 49.2 kJ/min and Arrhenius constant was 16.47 × 10^10^ min^−1^. The NaOH@GO-Fe_3_O_4_ catalyst could generate five times, with average biodiesel yield 86.6%. The physicochemical characterization of the biodiesel which produce under optimum condition had a good contract with the ASTM standard, therefore, producing biodiesel via NaOH@GO-Fe_3_O_4_ from WCO can performed in industrial scale due to low cost and high efficiency of catalyst.

## Data Availability

Data are available [from Sepideh Moradi Haghighi] with the permission of [Alireza Hemmati]. The data that support the findings of this study are available from the corresponding author, [Alireza Hemmati and, Hamidreza moghadamzadeh], upon reasonable request.
